# Retinol Stimulates Growth Factor Production and Extracellular Matrix-Related Marker Levels in Human Dermal Fibroblasts

**DOI:** 10.3390/biom16101530

**Published:** 2026-10-20

**Authors:** Murat Türkoğlu, Ali Türkan, Gülistan Öncü, Hakan Sevinç, Erkin Pekmezci

**Affiliations:** 1 R&D Center, Biota Laboratories, Sancaktepe, 34785 Istanbul, Türkiye; mturkoglu@biotalab.com (M.T.); aturkan@biotalab.com (A.T.); goncu@biotalab.com (G.Ö.); hsevinc@biotalab.com (H.S.); 2 Department of Dermatology, International Faculty of Medicine, Istanbul Medipol University, 34810 Istanbul, Türkiye

**Keywords:** retinol, human dermal fibroblast, growth factors, HAS-2, collagen, antiaging, wound healing

## Abstract

Retinol, a vitamin A derivative belonging to the retinoid family, is widely recognized for its anti-aging and wound-healing properties. In this study, we investigated the effects of retinol on the production of growth factors and major extracellular matrix (ECM) components in human dermal fibroblasts. Cells were treated with 0.05 and 0.5 μM retinol, and the levels of vascular endothelial growth factor (VEGF), fibroblast growth factor-2 (FGF-2), transforming growth factor-beta (TGF-β), hyaluronan synthase-2 (HAS-2), collagen type-I (COL-I), and collagen type-III (COL-III) were quantified using ELISA. Retinol treatment significantly increased the levels of VEGF, FGF-2 and TGF-β at both concentrations tested. Similarly, the levels of the ECM-related markers HAS-2, COL-I and COL-III were elevated following retinol exposure at both concentrations. Collectively, these findings indicate that retinol induces a coordinated pro-regenerative response in human dermal fibroblasts, characterized by enhanced extracellular matrix production, angiogenic signaling, hyaluronic acid synthesis, and tissue remodeling. These results further support the role of retinol as an antiaging and wound healing agent that promotes skin regeneration and maintenance.

## 1. Introduction

The skin performs a critical role as the primary defense barrier against various environmental threats such as solar ultraviolet (UV) radiation, other physical injuries, infections and chemical injuries. Apart from subcutaneous fat tissue, human skin is composed of two main layers. The outer layer is the epidermis, which basically comprises keratinocytes. The underlying layer, the dermis, contains fibroblasts; proteins such as collagen, elastin, fibronectin, and proteoglycans; and polysaccharides, also called glycosaminoglycans, such as hyaluronic acid (HA) and chondroitin sulfate. The protein and carbohydrate structures constitute the extracellular matrix (ECM). Collagen, making up approximately ninety percent of the skin’s dry weight, is the predominant protein [1] and supplies the dermis its structural stability and resilience [1,2]. The glycoproteins have functions in cell migration, adhesion and orientation, which are necessary for production of granulation tissue and other facets of wound healing. One of the basic roles of glycosaminoglycans is to bind water and give the skin its rubbery consistency. In aging skin, the glycosaminoglycan composition of the dermis changes, and water cannot be bound efficiently, leading to decreased skin distention [3].

Unlike many other organs, skin is repeatedly exposed to environmental stresses during the natural aging process. Therefore, aging of the skin can be classified into two specific types based on their underlying causes: Intrinsic or chronological aging, which occurs as a natural result of the flow of time, and extrinsic aging, which results from external factors, most notably UV radiation exposure [4]. Although underlying mechanisms differ, they share common molecular characteristics. The aging process in the dermis can predominantly be attributed to three specific molecular events: The breakdown of collagen fibers due to the increased activity of matrix metalloproteinases, reduced collagen production caused by compromised transforming growth factor-β (TGF-β) signaling, and the presence of an inflammatory microenvironment known as inflammaging [1]. The age-related alterations in the dermis bring various skin conditions, including increased fragility [5], impaired support for blood vessels [6], and compromised wound healing [7,8].

Fibroblasts and ECM affect each other reciprocally. Although dermal fibroblasts play an essential role in synthesizing, organizing, and maintaining the ECM, the composition, organization, and mechanical properties of the ECM they inhabit also can modulate the phenotype and behavior of fibroblasts [1]. Like in the aging process, fibroblasts and ECM play a major role also in wound healing, which mainly consists of three phases. After the initial inflammation stage, the second stage of wound healing is characterized by proliferation. This stage usually lasts from injury up to five to seven days, generally overlapping the inflammation stage. Characteristic changes include capillary growth and granulation tissue formation, fibroblast proliferation with collagen synthesis, and increased macrophage and mast cell activity. Growth factors, especially TGF-β, acting in concert with the ECM molecules, stimulate fibroblasts to proliferate and migrate into the wound space [2]. Fibroblast growth factor-2 (FGF-2) may initiate angiogenesis during the first three days of wound repair, whereas vascular endothelial growth factor (VEGF) is critical for angiogenesis during the formation of granulation tissue on days four to seven [9]. The third stage of wound healing is defined by maturation. This long phase of contraction, tissue remodeling, and increasing tensile strength may last up to a year [10].

Retinol, a derivative of vitamin A and classified among retinoids, is known for its beneficial effects as an antiaging and wound-healing agent [11,12,13]. The mechanisms of retinol action may involve the activation of nuclear retinoic acid receptors (RARs) and retinoid X receptors (RXRs), which regulate gene transcription and cell differentiation, and re-prescribe the cellular phenotype. When retinol is applied topically to human skin, it can penetrate the skin and undergo sequential conversion to retinaldehyde and then to retinoic acid, the active metabolite [1,14]. Retinol has demonstrated efficacy in improving the visual manifestations of both intrinsic and extrinsic aging, such as wrinkles, fine lines, and irregular pigmentation [11,15,16,17]. Topical retinoids, besides improving epidermal thickness, have also shown a remarkable increase in the proliferation of endothelial cells and blood vessels in the papillary dermis in vivo [18,19,20]. Analysis of published RNA-Seq data from wounds of mice at different time points confirmed the activation of retinol metabolism–associated genes in the proliferative phase of wound healing, especially in fibroblasts [13,21]. Serum retinol levels were found decreased in patients with burn wounds or chronic leg ulcers [13]. In some animal studies, topical retinoid treatment improved repair in healing-impaired mice or rats [22,23]. In a human study, short-contact treatment of chronic leg ulcers with tretinoin increased granulation tissue formation in male and female patients [24].

The accurate cellular and molecular mechanisms underlying the abovementioned beneficial effects of retinoids are still not comprehensively understood. Additionally, when compared to retinoic acid, there is a paucity of research investigating the molecular foundations of the effects of topical retinol in human skin [1]. Also, although there are some transcriptional-level in vitro and some in vivo studies concerning the antiaging and/or wound healing effects of retinoids, translational, protein-level studies are very scarce. In this study we investigated the protein-level effects of retinol on some of the main growth factors, an anabolic enzyme, and main dermal structural proteins in a human dermal fibroblast line to reveal their probable contributions to its antiaging and wound healing effects.

## 2. Materials and Methods

### 2.1. Preparation of Retinol

A 40 mM stock solution of retinol (All-trans-Retinol, 95%, Thermo Scientific, Waltham, MA, USA) was freshly prepared in dimethyl sulfoxide (DMSO; Applichem, Arheilgen, Germany) for each experiment. The solution was thoroughly mixed using a vortex mixer until completely homogenized and then passed through a 0.22 µm sterile filter. Due to the photosensitivity of retinol, the tubes were wrapped in aluminum foil to protect the solution from direct light during preparation and handling. The stock solution was freshly prepared immediately before each experiment, and the required working concentrations were prepared in culture medium immediately before treatment. All solutions were protected from direct light during preparation and use.

### 2.2. Cell Culture

Human fibroblasts, CCD-1135Sk (Cellosaurus accession number: CVCL_2370; ATCC CRL-2691), were purchased from the American Type Culture Collection (ATCC, Manassas, VA, USA). The cells were cultured in Iscove’s Modified Dulbecco’s Medium (IMDM; Pan-Biotech, Aidenbach, Germany) supplemented with 15% fetal bovine serum (FBS; Sigma-Aldrich, Burlington, MA, USA), 4 mM L-glutamine (Gibco, Thermo Fisher Scientific, Waltham, MA, USA), and 1% penicillin–streptomycin (Pan-Biotech, Aidenbach, Germany). The cell line was incubated at 37 °C in a humidified atmosphere of 95% air and 5% CO_2_ and was regularly passaged at approximately 80% confluence.

### 2.3. Cell Viability Assay

The effects of retinol on CCD-1135Sk cell viability were assessed using the 2,3-bis(2-methoxy-4-nitro-5-sulfophenyl)-2H-tetrazolium-5-carboxanilide (XTT) assay (Roche Diagnostics, Mannheim, Germany) in accordance with the manufacturer’s instructions. Cells were seeded into 96-well plates at a density of 10^4^ cells per well and allowed to adhere overnight. Subsequently, the cells were treated with retinol at concentrations ranging from 0.01 to 10 µM for 24, 48, and 72 h. A vehicle control was included to assess any potential cytotoxic effects of the solvent used to dissolve retinol. Following treatment, 50 µL of XTT labeling mixture was added to each well, and the plates were incubated for 4 h at 37 °C. Absorbance was measured at 490 nm with a reference wavelength of 650 nm using a microplate reader (Bio-Rad, Hercules, CA, USA). Cell viability was expressed as a percentage relative to the untreated control.

### 2.4. Cell Proliferation Assay

Cell proliferation was evaluated using the 5-Bromo-2′-deoxyuridine (BrdU) Cell Proliferation Assay (Cat. No. 2750-M, Sigma-Aldrich/Millipore, St. Louis, MO, USA) in accordance with the manufacturer’s protocol. Briefly, cells were seeded into 96-well plates at a density of 2 × 10^5^ cells/mL and exposed to retinol at concentrations ranging from 0.01 to 1 µM for 24 or 48 h. BrdU was incorporated during the final 4 h of each treatment period. Subsequently, the cells were fixed and the DNA was denatured, followed by detection of BrdU incorporation using an anti-BrdU monoclonal antibody and an HRP-conjugated secondary antibody. Color development was achieved using TMB substrate, and absorbance was measured at 450 nm with a microplate reader (Bio-Rad, Hercules, CA, USA). BrdU incorporation was quantified as a measure of cellular proliferation.

### 2.5. Treatment of CCD-1135Sk Cells with Retinol

The CCD-1135Sk cells were seeded at a density of 10^5^ cells/well in 6-well plates and incubated overnight to allow attachment. For VEGF, FGF-2 and TGF-β analyses, cells were incubated in low-serum medium and treated with 0.05 and 0.5 μM retinol for 24 h. Hyaluronic acid synthase-2 (HAS-2) levels were tested after 24 h under standard culture conditions, whereas collagen type-I (COL-I) and collagen type-III (COL-III) levels were tested after 48 h under the same condition. At the end of the incubation period, cell culture supernatants were collected and centrifuged to remove cell debris. The supernatants were aliquoted and stored at −80 °C until further analysis.

### 2.6. Enzyme-Linked Immunosorbent Assay (ELISA)

The protein concentrations were measured using a bicinchoninic acid assay kit (BCA, Thermo Scientific, Waltham, MA, USA) for normalization across samples. Levels of VEGF, FGF-2, TGF-β, HAS-2, COL-I, and COL-III were measured using ELISA kits (YL Biont, Shanghai, China) according to the manufacturer’s protocols. Absorbance was measured at 450 nm using a microplate reader (Bio-Rad, USA). All measurements were performed in triplicate.

### 2.7. Statistical Analysis

All experiments were conducted in triplicate with independent samples, and the results are expressed as mean ± standard deviation (SD). Statistical significance was determined using Student’s *t*-test, with *p* < 0.05 considered significant. Data analysis was performed using OriginPro (version 10.2.5; OriginLab Corporation, Northampton, MA, USA).

## 3. Results

### 3.1. Effect of Retinol Treatment on the Viability of Human Dermal Fibroblast Cells

**Figure 1:**
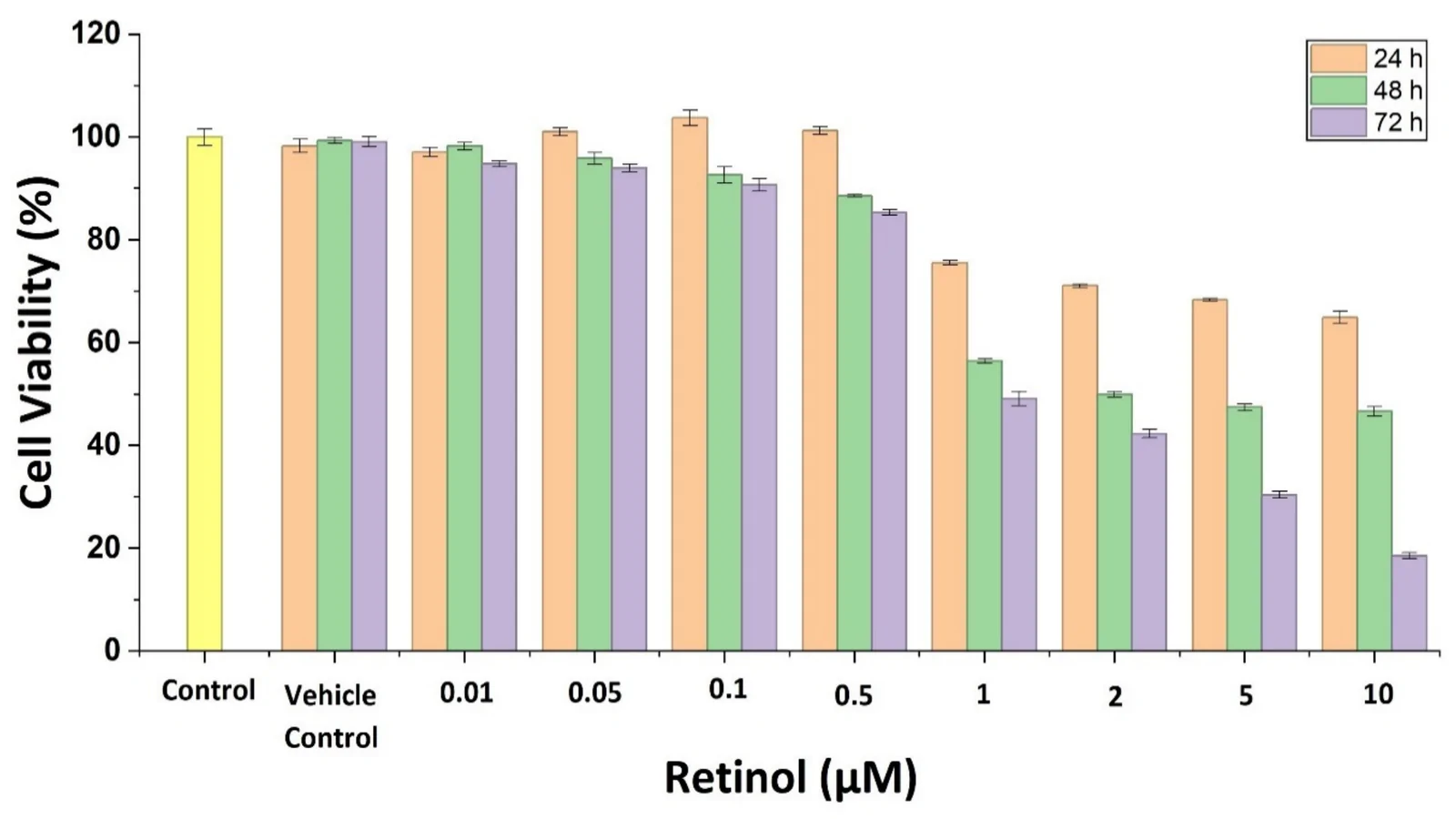
Effect of retinol on the viability of human dermal fibroblast cells following 24, 48, and 72 h of exposure. Human dermal fibroblast cells were treated with increasing concentrations of retinol (0.01–10 µM) for 24 h (orange bars), 48 h (green bars), and 72 h (purple bars). Untreated control cells and vehicle-treated control cells were included for comparison. Cell viability was determined using the XTT assay and expressed as a percentage relative to the untreated control (100%). Data are presented as mean ± SD from three independent experiments (*n* = 3).

The effects of retinol on human dermal fibroblast viability were evaluated at concentrations ranging from 0.01 to 10 µM following 24, 48, and 72 h of exposure (Figure 1). Low retinol concentrations (0.01–0.5 µM) had minimal effects on cell viability, which remained comparable to the untreated and vehicle controls throughout the study period. At these concentrations, cell viability generally remained above 85%, with values close to 100% at 24 h. In contrast, higher retinol concentrations (≥1 µM) reduced cell viability in both a concentration- and time-dependent manner. After 24 h of treatment, cell viability decreased to approximately 76%, 71%, 68%, and 65% at 1, 2, 5, and 10 µM retinol, respectively. The inhibitory effect became more pronounced following prolonged exposure. At 48 h, viability further declined to approximately 56%, 50%, 48%, and 47% at the respective concentrations, while after 72 h viability was reduced to approximately 49%, 42%, 30%, and 18%. No notable difference was observed between the untreated and vehicle control groups, indicating that the vehicle itself did not affect cell viability. Overall, these findings demonstrate that retinol exerts a clear dose- and time-dependent inhibitory effect on the viability of human dermal fibroblasts, whereas concentrations of 0.5 µM and below were generally well tolerated under the experimental conditions. Therefore, two retinol concentrations, 0.05 µM and 0.5 µM, were selected for subsequent studies.

### 3.2. Effect of Retinol Treatment on the Proliferation of Human Dermal Fibroblast Cells

**Figure 2:**
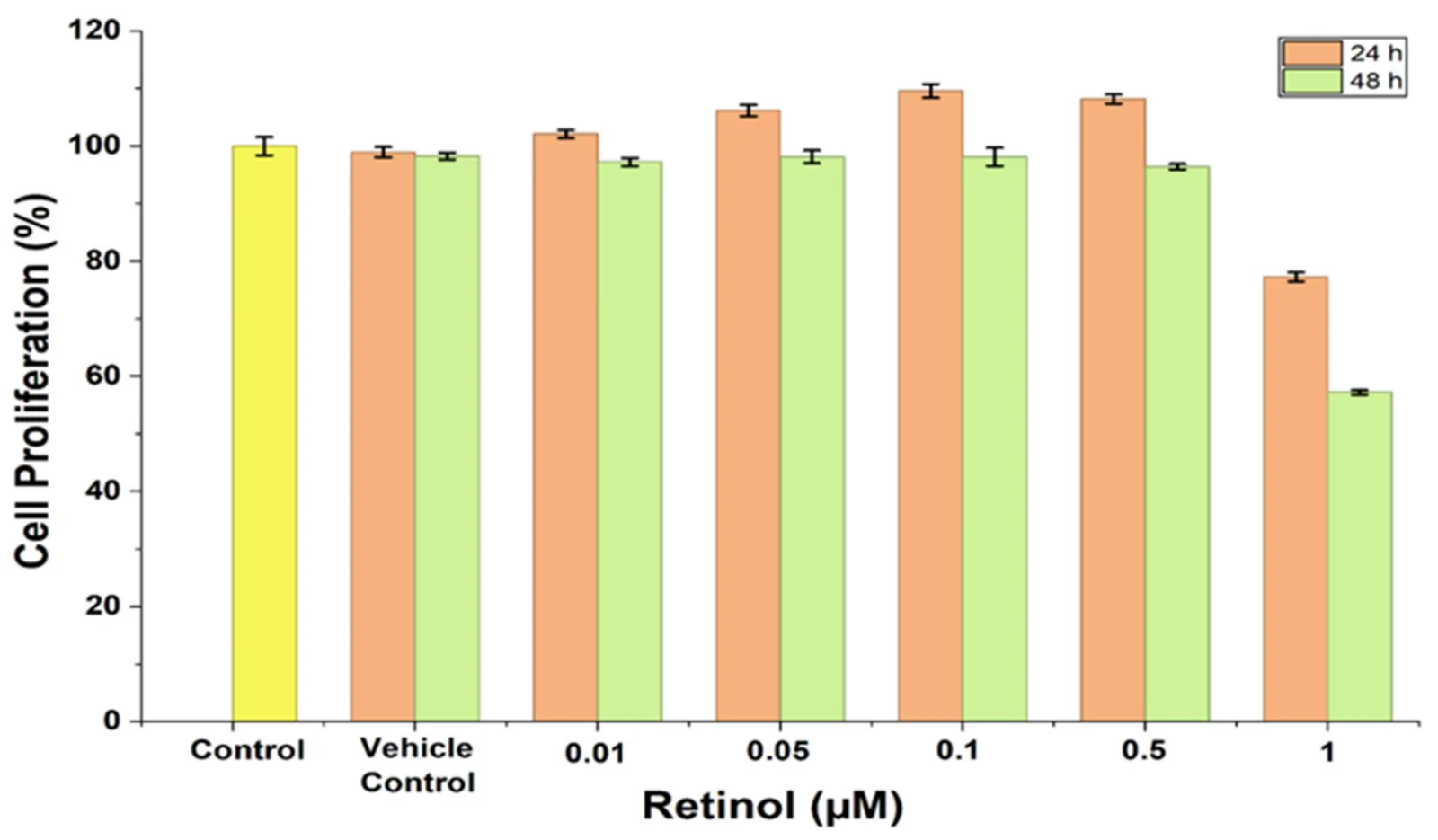
Effect of retinol on the proliferation of human dermal fibroblast cells following 24 and 48 h of exposure. Human dermal fibroblast cells were treated with increasing concentrations of retinol (0.01–1 µM) for 24 h (pink bars) and 48 h (green bars). Untreated control cells and vehicle-treated control cells were included for comparison. Cell viability was determined using the BrDU assay and expressed as a percentage relative to the untreated control (100%). Data are presented as mean ± SD from three independent experiments (*n* = 3).

To evaluate the effect of retinol on cell proliferation, human dermal fibroblast (HDF) cells were treated with 0.01–1 μMretinol for 24 and 48 h (Figure 2). Treatment with concentrations up to 0.5 μM maintained cell proliferation at levels comparable to controls, whereas 1 μM retinol significantly reduced proliferation to 77% at 24 h and 57% at 48 h. Accordingly, 0.05 μM and 0.5 μM were selected for ELISAs. These two working concentrations represent a 10-fold dose range and fall within the optimal non-inhibitory proliferation range. The lower concentration (0.05 μM) was selected as an effective physiological dose, whereas the higher concentration (0.5 μM) represented the maximum concentration that maintained normal cell proliferation before the significant reduction observed at 1 μM.

### 3.3. Effect of Retinol on the Production of Growth Factors

**Figure 3:**
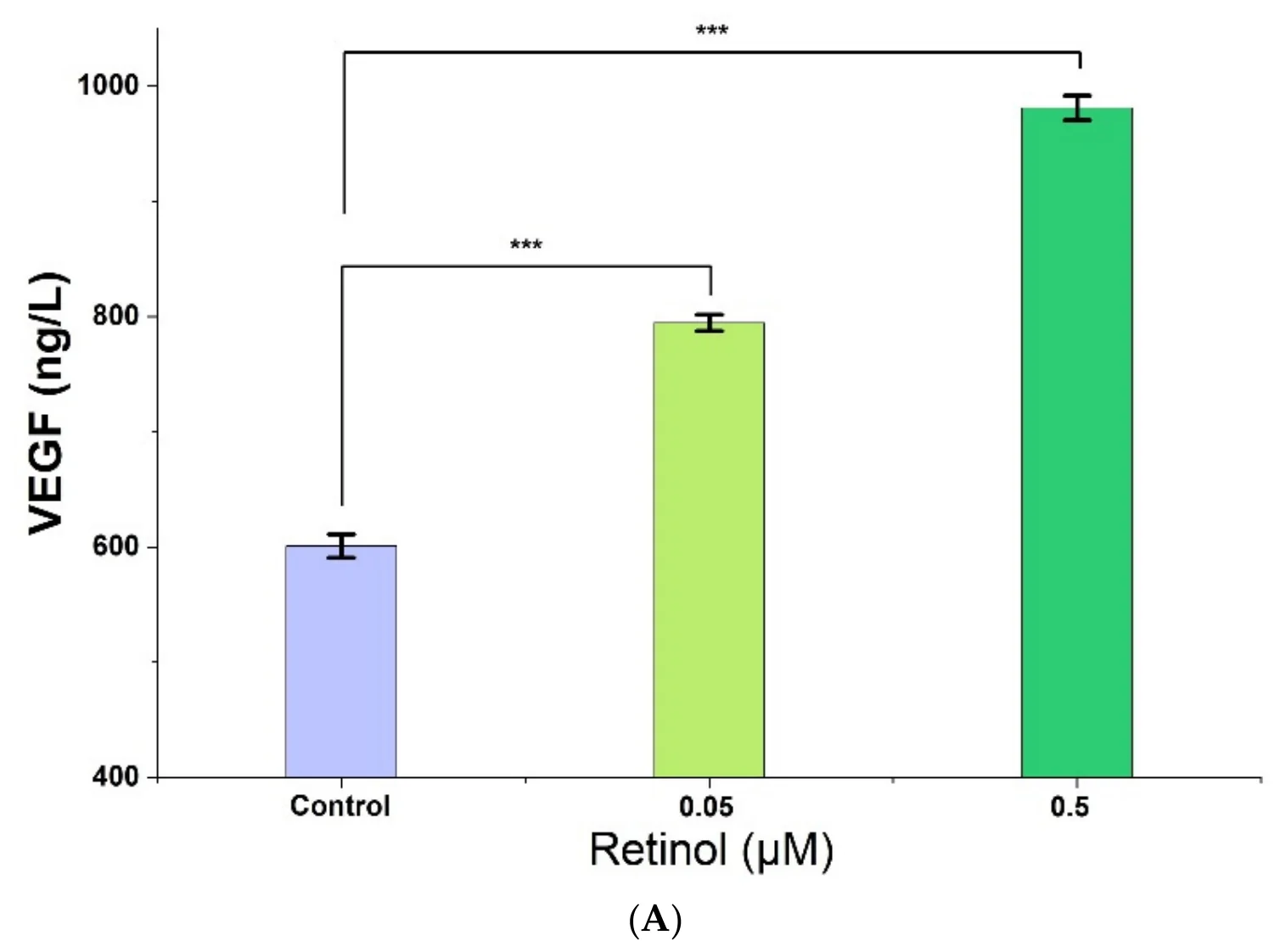

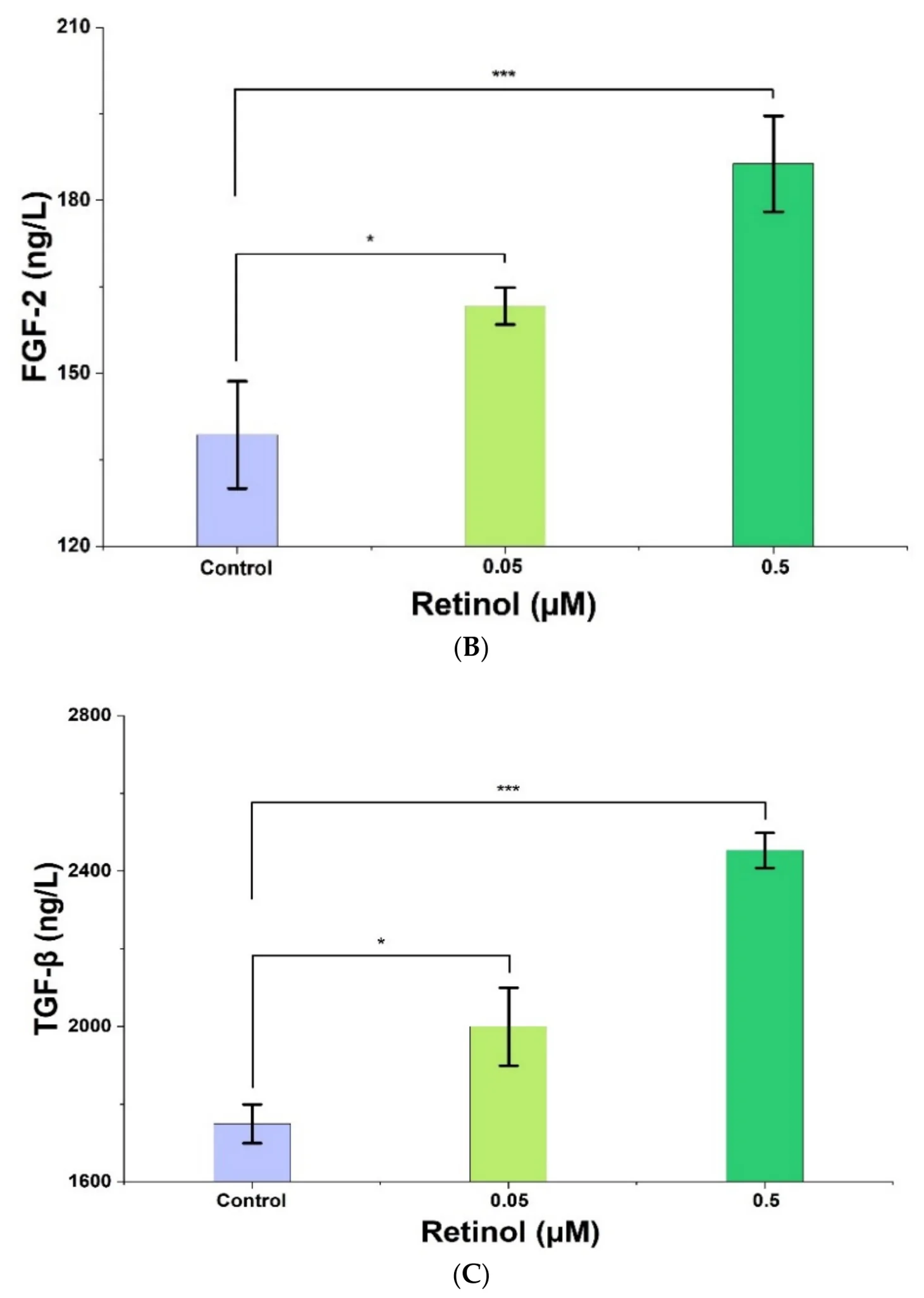
Effect of retinol treatment on growth factor protein levels in human dermal fibroblasts. Protein levels of (**A**) VEGF, (**B**) FGF-2, and (**C**) TGF-β were determined in human dermal fibroblasts treated with retinol (0.05 and 0.5 μM) and compared to untreated control cells. Experiments were performed as described in the Materials and Methods section. Data are presented as mean ± SD (*n* = 3). Statistical significance is indicated as * *p* < 0.05 and *** *p* < 0.001 compared to the control group.

To investigate the effect of retinol on growth factor production, the levels of VEGF, FGF-2, and TGF-β were assessed in human dermal fibroblasts following treatment with retinol. As shown in Figure 3A, retinol treatment significantly increased VEGF production compared to the untreated control group. VEGF levels increased from approximately 600 ng/L in the control group to 795 ng/L and 980 ng/L following treatment with 0.05 and 0.5 μM retinol, respectively. Both retinol concentrations produced a highly significant increase in VEGF secretion (*p* < 0.001).

Similarly, FGF-2 production was enhanced by retinol treatment (Figure 3B). FGF-2 levels increased from approximately 140 ng/L in the control group to 162 ng/L and 186 ng/L after treatment with 0.05 and 0.5 μM retinol, respectively. The increase observed at 0.05 μM was statistically significant (*p* < 0.05), whereas treatment with 0.5 μM retinol resulted in a more pronounced increase (*p* < 0.001) compared to the control group.

A comparable trend was observed for TGF-β production (Figure 3C). TGF-β levels increased from approximately 1750 ng/L in untreated cells to 2000 ng/L and 2450 ng/L following exposure to 0.05 and 0.5 μM retinol, respectively. The increase at 0.05 μM was significant (*p* < 0.05), while treatment with 0.5 μM retinol induced a higher significant elevation in TGF-β levels (*p* < 0.001) relative to the control group.

### 3.4. Effect of Retinol on the Production of HAS-2

**Figure 4:**
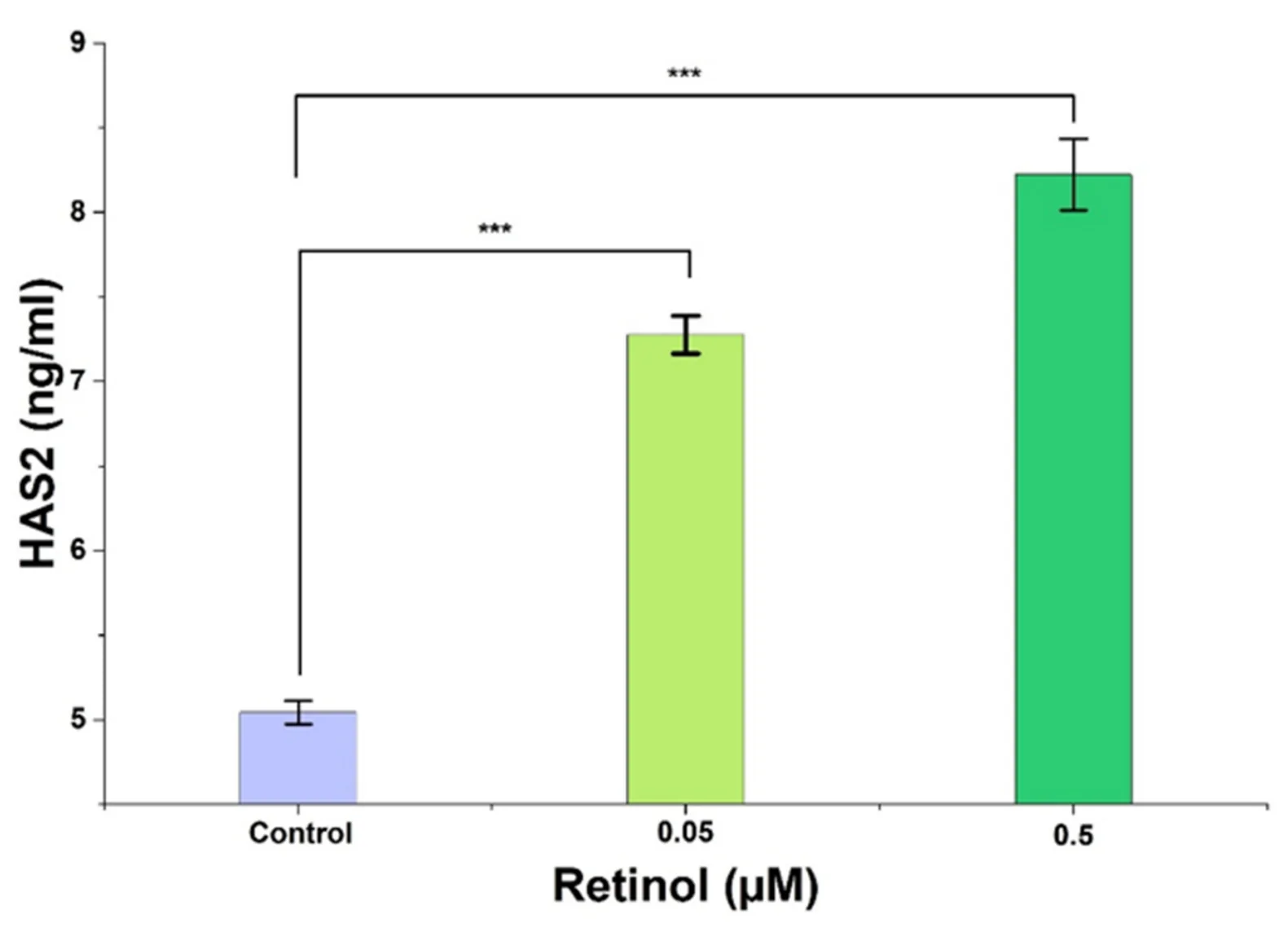
Effect of retinol treatment on HAS-2 levels in human dermal fibroblast cells. HAS-2 levels in cells treated with retinol (0.05 and 0.5 μM) compared to those in the untreated control group. Experiments were performed as described in the Materials and Methods section. Data are presented as mean ± SD (*n* = 3). Statistical significance is indicated as *** *p* < 0.001 compared to the control group.

The effect of retinol on anabolic HAS-2 enzyme levels in human dermal fibroblasts was evaluated following treatment with 0.05 and 0.5 μM retinol (Figure 4). Both retinol concentrations produced a highly significant increase in HAS-2 secretion (*p* < 0.001) compared to the control group.

### 3.5. Effect of Retinol on the Production of COL-I and COL-III

**Figure 5:**
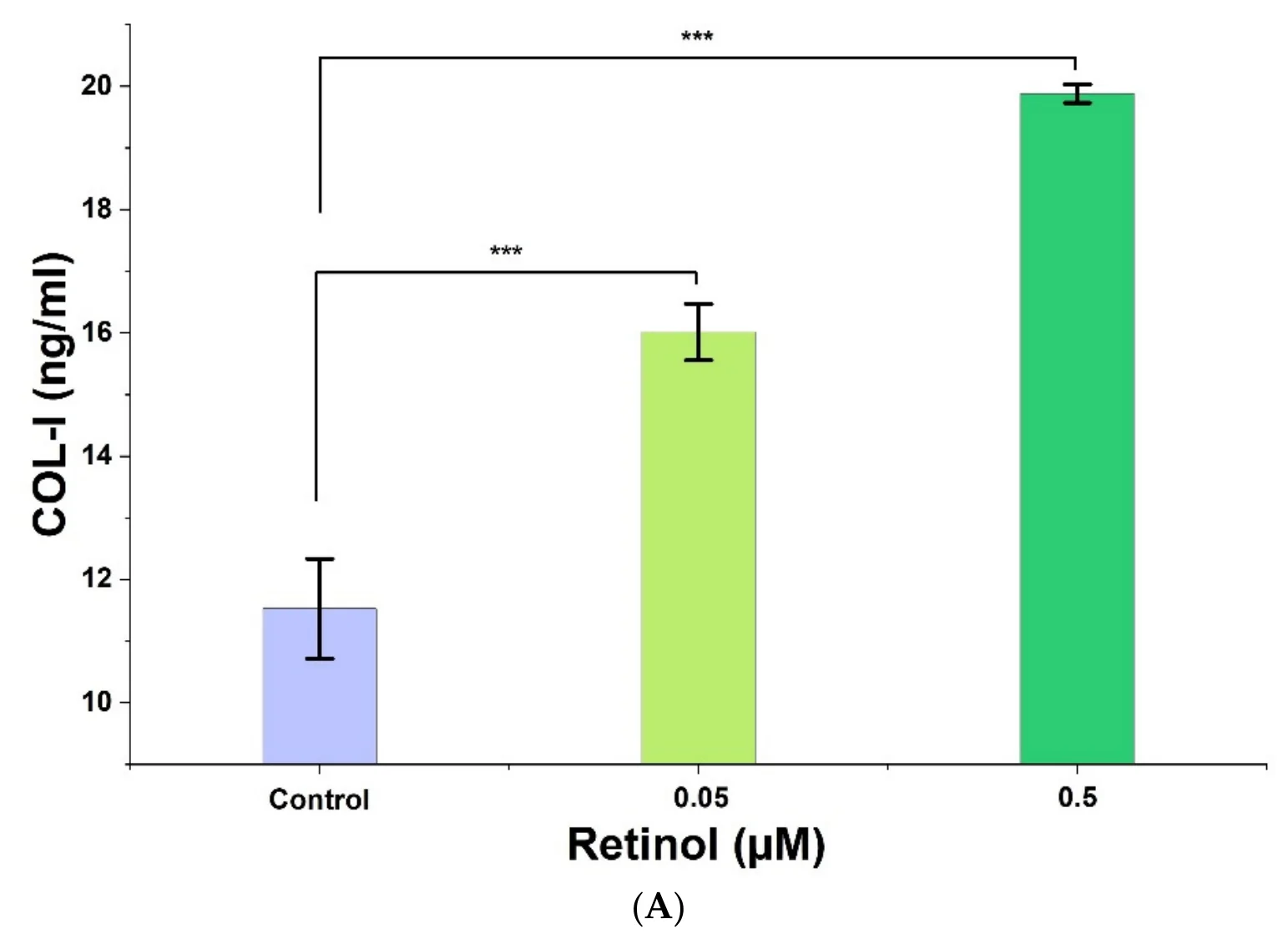

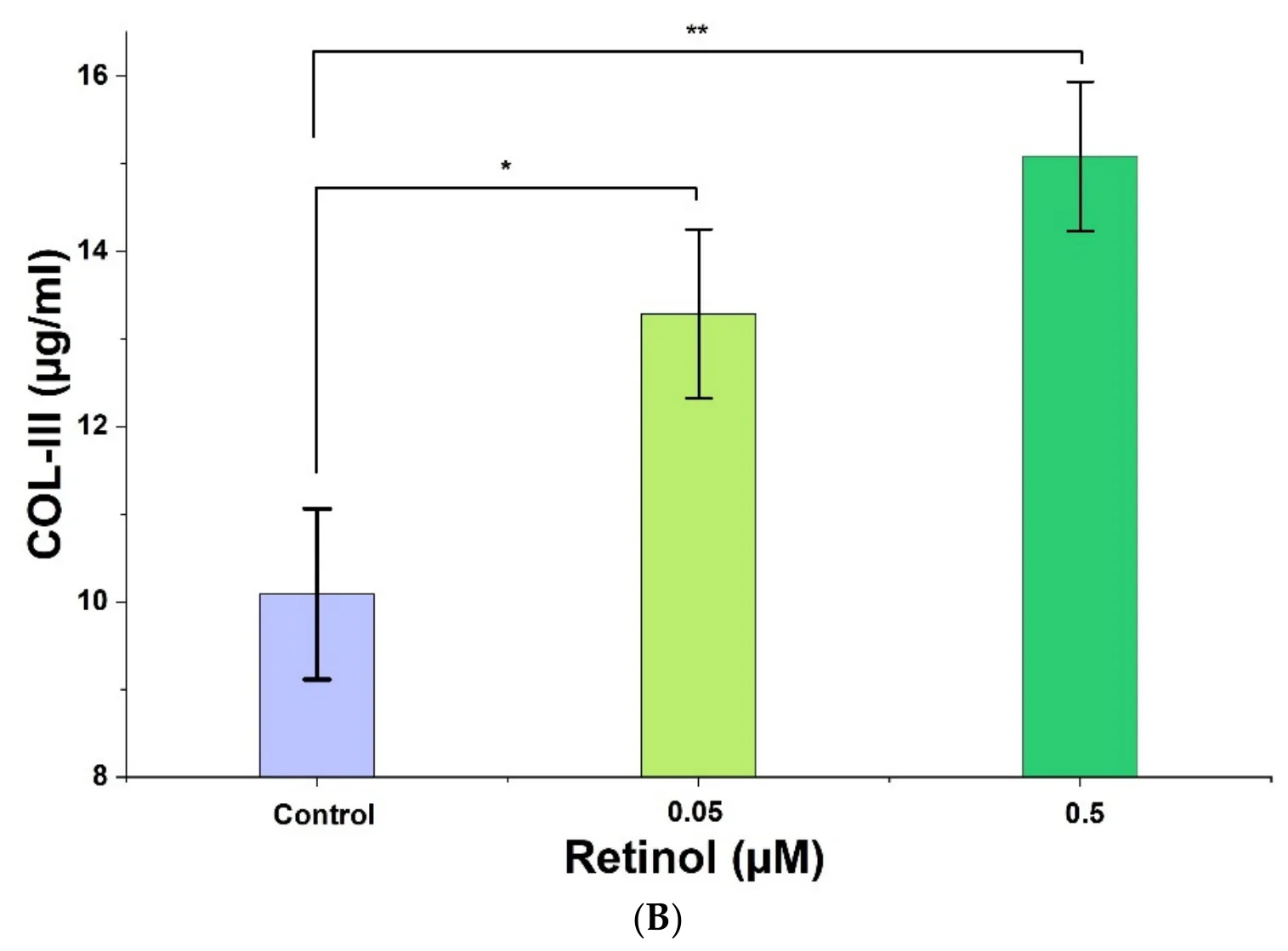
Effect of retinol treatment on collagen production in human dermal fibroblast cells. (**A**) COL-I and (**B**) COL-III levels in cells treated with retinol (0.05 and 0.5 μM) compared to the untreated control group. Experiments were performed as described in the Materials and Methods section. Data are presented as mean ± SD (*n* = 3). Statistical significance is indicated as * *p* < 0.05, ** *p* < 0.01, and *** *p* < 0.001 compared to the control group.

To evaluate the effect of retinol on ECM structural composition, the levels of COL-I and COL-III were measured in human dermal fibroblasts following treatment with 0.05 and 0.5 μM retinol. Retinol treatment significantly increased COL-I production compared to the untreated control group (Figure 5A). COL-I levels increased from approximately 11.5 ng/mL in the control group to 16.0 ng/mL and 19.9 ng/mL following treatment with 0.05 and 0.5 μM retinol, respectively. Both retinol concentrations resulted in a highly significant increase in COL-I levels (*p* < 0.001).

Similarly, retinol treatment significantly enhanced COL-III production (Figure 5B). COL-III levels increased from approximately 10.1 μg/mL in the control group to 13.3 μg/mL and 15.1 μg/mL after treatment with 0.05 and 0.5 μM retinol, respectively. The increase observed at 0.05 μM was statistically significant (*p* < 0.05), whereas treatment with 0.5 μM retinol produced a more pronounced increase (*p* < 0.01) compared to the control group.

## 4. Discussion

**Figure 6:**
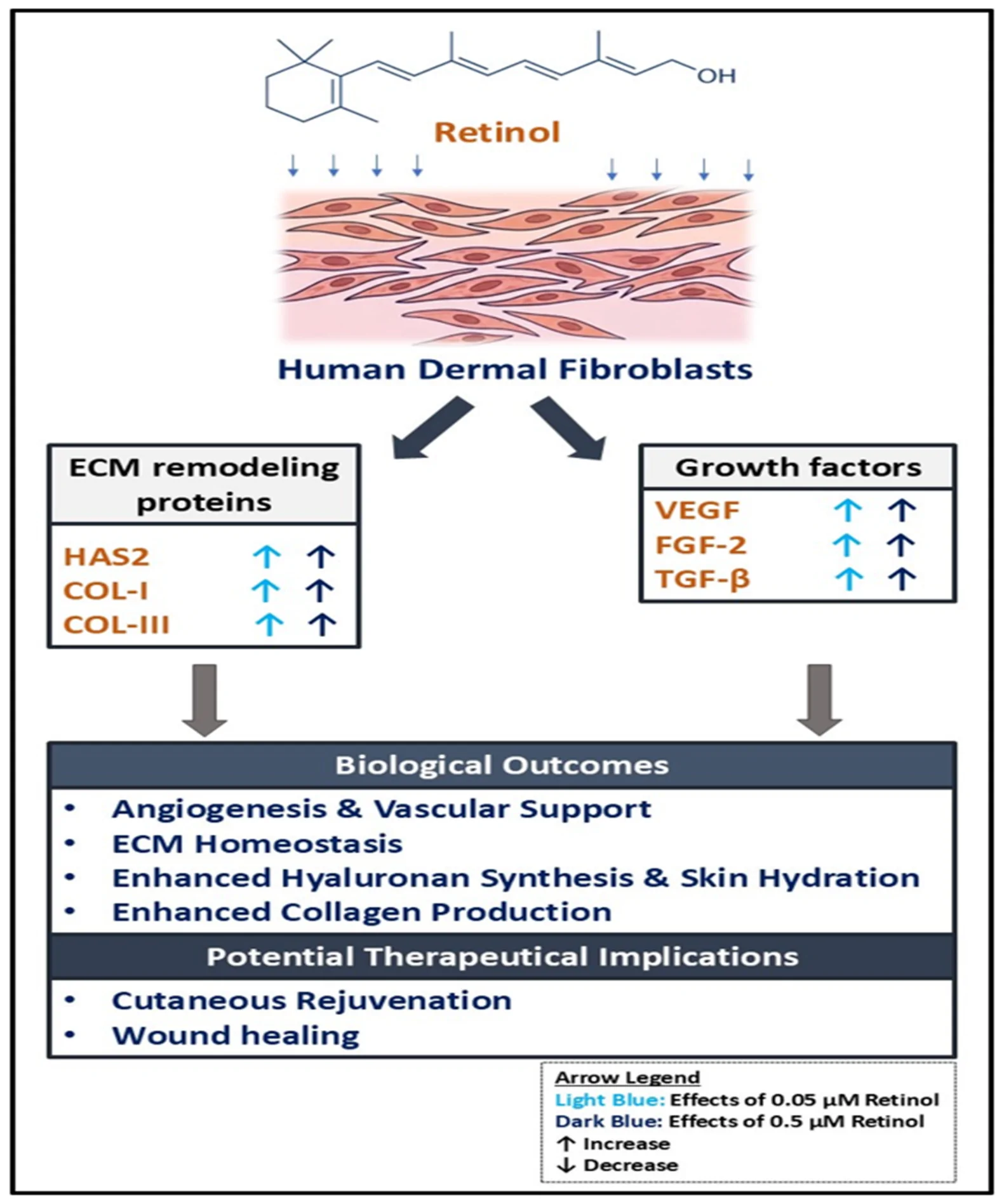
Proposed mechanisms underlying the biological effects and potential therapeutic implications of retinol in human dermal fibroblasts. Schematic flowchart illustrating the effects of retinol treatment on key growth factors (VEGF, FGF-2, and TGF-β) and ECM remodeling proteins (HAS-2, COL-I, and COL-III). Light blue arrows indicate the effects of 0.05 µM retinol, whereas dark blue arrows denote the effects of 0.5 µM retinol. Retinol-induced molecular responses are linked to biological processes leading to angiogenesis, ECM homeostasis, hydration, and collagen synthesis, suggesting potential implications on skin aging and tissue repair.

In the present study, retinol increased the protein levels of VEGF, FGF-2, TGF-β, HAS-2, COL-I and COL-III produced by dermal fibroblasts. Altogether, these findings suggest retinol induces a coordinated pro-regenerative fibroblast response entailing ECM production, angiogenic signaling, HA synthesis and tissue remodeling. These results are quite pertinent to both antiaging and wound healing physiologies, because dermal aging and impaired wound healing are both characterized by diminished collagen synthesis, altered ECM organization, impaired vascular support and reduced dermal hydration [1,11,18,25]. In Figure 6, the proposed molecular responses of human dermal fibroblasts to retinol treatment are summarized.

The increase in TGF-β is of paramount importance, as it is one of the major enhancers of dermal fibroblast activity and ECM synthesis. In normal human dermal fibroblasts, TGF-β increases the expression of COL-I, COL-III and fibronectin, suggesting a major role in connective tissue production [26]. Therefore, retinol-induced TGF-β elevation may provide a credible mechanism for restoration of fibroblasts’ anabolic activities in aging skin. In the wound healing context, TGF-β promotes fibroblast proliferation and migration, myofibroblast differentiation, wound contraction, and collagen deposition [26,27].

The increase in VEGF indicates that retinol-treated fibroblasts may increase angiogenic signaling. VEGF is an important pro-angiogenic factor in skin repair, stimulating endothelial cell migration, proliferation and new capillary formation [28]. Previously retinoic acid was demonstrated to increase VEGF expression in normal human dermal fibroblasts and to enhance angiogenesis through RAR-mediated mechanisms [29]. As the aged skin shows reduced vascularity and angiogenesis is essential for granulation tissue formation [28,29], the increase in VEGF may support both the antiaging and wound healing effects of retinol. FGF-2 is a mitogenic and angiogenic growth factor that promotes fibroblast proliferation, endothelial cell activity, and granulation tissue formation, which may contribute to efficient wound repair. In the context of aging, increased FGF-2 may offset the decline in fibroblast number and function. FGF-2 acts together with VEGF to support angiogenesis [30,31]. Therefore, the simultaneous increase in FGF-2 and VEGF may indicate that retinol generates a promising environment for increased vascularization.

The major water-binding molecule in the ECM is HA, and it also enhances cell migration as well as dermal hydration. Skin aging is associated with loss and altered distribution of HA, leading to dryness, reduced turgor and diminished flexibility. In wound healing, HA supplies an ECM, easing fibroblast and keratinocyte migration, and supports granulation tissue organization [32]. Previously it was shown that retinol can increase HAS gene expressions and HA production in human skin models and human skin in vivo [33]. The HAS-2 is one of the main enzymes responsible for HA synthesis [32], and its increase in the present study suggests that retinol may boost the hydration of ECM and enhance the migration of fibroblasts.

The concurrent increase in COL-I and COL-III may be regarded as direct evidence of retinol-driven anabolic activity in the ECM. COL-I is the major structural collagen of mature dermis, and it is responsible for tensile strength. COL-III constitutes a more fragile and elastic network and is notable in younger connective tissue and early wound matrix [1,34]. Therefore, while increased COL-I may indicate an improved dermal structural strength, the increase in COL-III may suggest a more regenerative matrix response. Clinically, retinol was shown to increase procollagen-I or COL-I and ameliorate fine wrinkling in naturally aged skin, making the collagen findings in this study consistent with reported antiaging effects [18]. In wound healing, although the balance between COL-I and COL-III is biologically important and the altered COL-I/COL-III ratio may be associated with pathological scarring [34,35], the simultaneous increase in either collagen type in our study may suggest that retinol both reinforces initial matrix formation and later structural maturation.

The selection of retinol concentrations in our fibroblast cell culture models was grounded both in the viability of the cells in the first 72 h, and in the established in vivo percutaneous absorption efficiency of topical retinoids. The increased viability of the cells in the first 24 h, especially between 0.05 and 0.5 µM concentrations, is probably due to the increased proliferation caused by retinol, which is supported by the cell proliferation assay also (Figure 2). Due to the stratum corneum barrier, only a fraction of topically applied formulations penetrates into the viable dermis. Although the 0.05 μM retinol is considered the initial effective concentration used in cosmetic preparations [14,36], the 0.5 μM concentration of retinol represents the physiological steady-state level achieved in the dermis following the application of mid-to-high strength (0.5%) clinical formulations [37]. The effects of retinol on the three proteins in our study, TGF-β, FGF-2 and COL-III, were dose-dependent as expected. But, unexpectedly, its effects on VEGF, HAS-2 and COL-I were not dose-dependent and were highest even in the low-strength (0.05 µM) concentration, which may mean that the enhancing power of retinol is faster in the second group of proteins. The outcomes obtained by ELISA are important, as through this method, it is verified that the transcriptional reactions initiated by the RAR/RXR complexes successfully surpassed all post-transcriptional and translational steps, resulting in the active synthesis and extracellular secretion of these functional proteins [38,39]. Although retinol has already been reported in the literature for its antiaging and wound-healing properties, to date there is no specific report connecting these properties collectively to the individual markers (proteins) used in this study. Therefore, the novelty of this study is the revelation of the association between the clinical properties and the specific biomarkers that may generate those effects. But, as a limitation of the study, we should state that dermal fibroblast monoculture cannot fully reproduce the complexity of skin, where keratinocytes, endothelial cells, immune cells, and the organization of ECM interact dynamically [1]. Therefore, the relevant findings should be validated by future studies with co-culture systems or skin-equivalent cultures. Nevertheless, these results in general support an integrated model in which retinol activates dermal fibroblasts, forming a regenerative phenotype. In antiaging, this profile resists major characteristics of aging skin, such as collagen loss, reduced fibroblast activity, decreased HA content and compromised vascular support. In wound healing, the same profile supports the main biological processes of repair, such as angiogenesis, matrix formation, and remodeling.

## 5. Conclusions

The increases in VEGF, FGF-2, TGF-β, HAS-2, COL-I and COL-III supply consistent molecular evidence that retinol fosters a regenerative response in dermal fibroblasts. These findings reinforce the role of retinol as an antiaging agent, which can restore ECM homeostasis, improve hydration and enhance angiogenesis. These outcomes also have importance for wound healing, where coordinated activations of growth factor signaling, HA synthesis, vascular support and collagen production are required for effective tissue repair. The present results, besides giving evidence for its mechanism of actions, provide a strong basis for further investigations of retinol in cutaneous rejuvenation and wound healing models.

## Data Availability

The original contributions presented in this study are included in the article. Further inquiries can be directed to the corresponding author.

## Abbreviations

The following abbreviations are used in this manuscript:

COL-I	collagen type I
COL-III	collagen type III
ECM	extracellular matrix
FGF-2	fibroblast growth factor-2
HA	hyaluronic acid
HAS-2	hyaluronan synthase 2
TGF-β	transforming growth factor beta
VEGF	vascular endothelial growth factor
